# Good Cop, Bad Cop: Profiling the Immune Landscape in Multiple Myeloma

**DOI:** 10.3390/biom13111629

**Published:** 2023-11-07

**Authors:** Niyati Seshagiri Sharma, Bibha Choudhary

**Affiliations:** 1 Institute of Bioinformatics and Applied Biotechnology (IBAB), Electronic City, Bengaluru 560100, India; niyati0sharma@gmail.com; 2 Manipal Academy of Higher Education (MAHE), Manipal 576104, India

**Keywords:** multiple myeloma, tumor microenvironment, hematopoiesis, immune profiling, immunotherapy

## Abstract

Multiple myeloma (MM) is a dyscrasia of plasma cells (PCs) characterized by abnormal immunoglobulin (Ig) production. The disease remains incurable due to a multitude of mutations and structural abnormalities in MM cells, coupled with a favorable microenvironment and immune suppression that eventually contribute to the development of drug resistance. The bone marrow microenvironment (BMME) is composed of a cellular component comprising stromal cells, endothelial cells, osteoclasts, osteoblasts, and immune cells, and a non-cellular component made of the extracellular matrix (ECM) and the liquid milieu, which contains cytokines, growth factors, and chemokines. The bone marrow stromal cells (BMSCs) are involved in the adhesion of MM cells, promote the growth, proliferation, invasion, and drug resistance of MM cells, and are also crucial in angiogenesis and the formation of lytic bone lesions. Classical immunophenotyping in combination with advanced immune profiling using single-cell sequencing technologies has enabled immune cell-specific gene expression analysis in MM to further elucidate the roles of specific immune cell fractions from peripheral blood and bone marrow (BM) in myelomagenesis and progression, immune evasion and exhaustion mechanisms, and development of drug resistance and relapse. The review describes the role of BMME components in MM development and ongoing clinical trials using immunotherapeutic approaches.

## 1. Introduction

**Figure 1:**
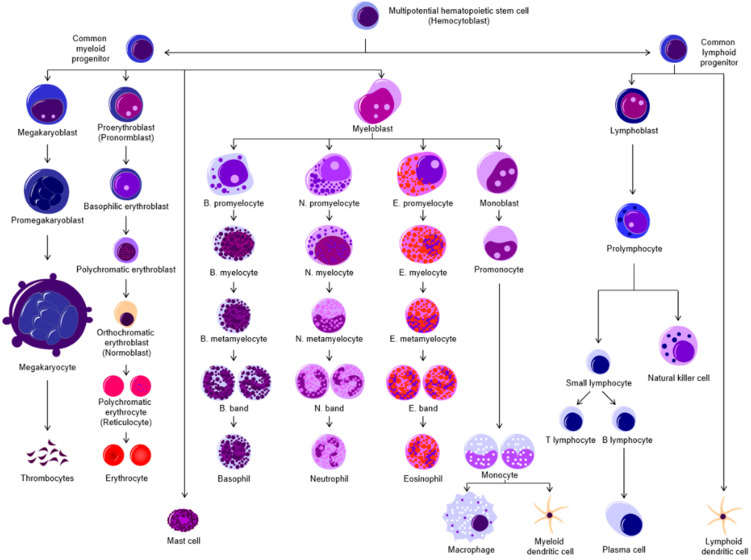
Hematopoiesis is the process by which all the blood cell types are produced through differentiation from increasingly lineage-committed precursor cells in the adult BM. Broadly, the cells can be classified as myeloid or lymphoid in origin. The myeloid lineage gives rise to thrombocytes (platelets), erythrocytes, granulocytes (mast cells, basophils, neutrophils, eosinophils), macrophages, and myeloid-derived dendritic cells (DCs). The lymphoid lineage produces T- and B-lymphocytes, natural killer (NK) cells, and lymphoid DCs.

B-cell development and maturation is a tightly regulated process. Throughout adult life, the bone marrow (BM) serves as the primary hotspot for a finite number of pluripotent hematopoietic stem cells (HSCs) to sequentially give rise to all the blood cell types through a process called hematopoiesis (Figure 1).

Immature plasma cells (PCs) originate from the lymphoid progenitor lineage [1,2], express a surface B-cell immunoglobulin receptor (BCR), and move to secondary lymphoid organs [3,4]. Here, the B-cells start to mature while remaining non-proliferative and transcriptionally dormant but primed for antigen recognition through the toll-like receptors (TLR) or the BCR [3,5]. B-cell activation can then occur in a T-cell-independent or dependent manner to generate acute or long-term immunity. The latter process involves complex activation by cytokines and affinity maturation in the germinal center of the lymph nodes. During activation and differentiation, B-cells are equipped to express a host of antibodies through the physiological process of class-switch recombination and somatic hypermutation [3,5,6]. However, these processes are highly error-prone and can cause the accumulation of genomic changes such as chromosomal translocations and aneuploidies [3,5]. Such translocations can place proto-oncogenes under the control of powerful enhancers in the Ig gene locus which can create aberrant PCs that can establish multiple myeloma (MM) precursor conditions such as monoclonal gammopathy of uncertain significance (MGUS) and smoldering MM (SMM) [5,7,8,9].

**Figure 2:**
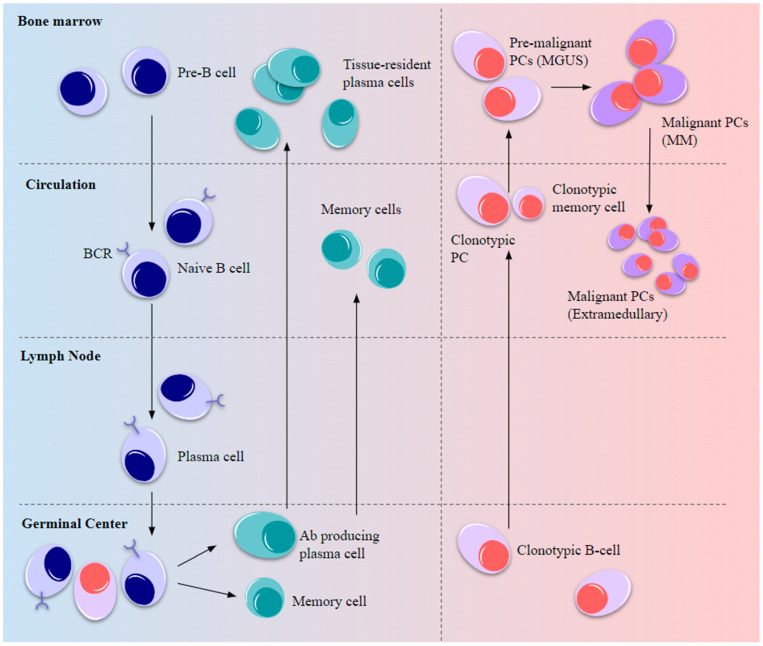
Normal B-cell development and myelomagenesis. Upon BCR activation by antigenic stimulus, B-cell precursors migrate from the BM to peripheral lymph nodes to elicit a short-term immune response and the remaining cells differentiate into long-lived circulating plasma and memory cells, eventually becoming resident cells in the tissue niches. Some of the post-germinal-center B-cells carrying oncogenic mutations enter the circulation as plasmablasts and memory B-cells that migrate to the BM as pre-malignant cells to establish MGUS. Further advantageous oncogenic mutations and the favorable BMME drive malignancy and MM development after which the cells become niche-independent and re-enter the circulation, resulting in extramedullary plasmacytoma.

In MM, a clonal subset of these transformed post-germinal-center B-cells undergoes malignant proliferation in the favorable microenvironment of the BM (Figure 2) to produce abnormal levels of Igs that are secreted into the serum and urine and termed as Bence-Jones proteins [9,10]. Monoclonal antibody accumulation in the BM can cause anemia, hypercalcemia and lytic bone lesions with accompanying bone pain and fractures, and impaired kidney function leading to renal failure [6,8,9]. These bone- and organ-damaging events are characteristic features of MM, often abbreviated as “CRAB” which stands for hyperCalcemia, Renal involvement, Anemia, and Bone lesions [8,9]. 

### Epidemiology, Diagnosis, and Cytogenetics in Multiple Myeloma

**Figure 3:**
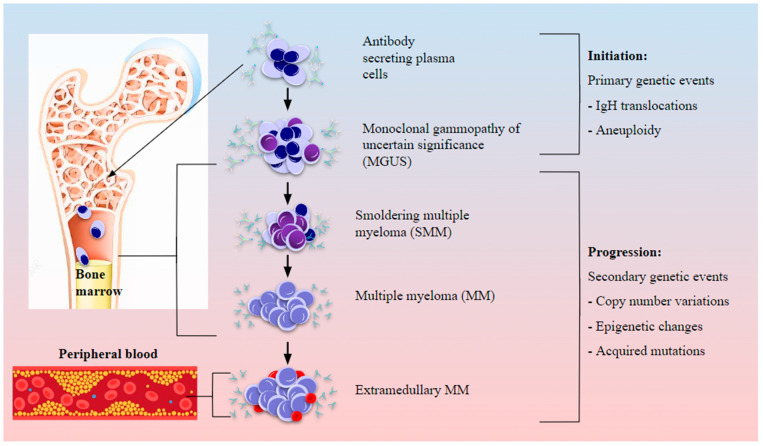
Cytogenetic abnormalities and involvement of the BMME in myeloma initiation and progression. Abnormal PCs in the BM harbor chromosomal abnormalities such as translocations and aneuploidy which could establish the precursor condition of monoclonal gammopathy of uncertain significance (MGUS). BM involvement and modification of the immune landscape by myeloma cells sets off myeloma genesis, first as smoldering MM (SMM), and with the accumulation of secondary genetic abnormalities such as CNVs and epigenetic changes as well as oncogenic driver mutations, active MM is established which eventually progresses to aggressive extramedullary plasmacytoma upon bone lysis and egress from the marrow to peripheral blood.

As of 2020, 176,404 new cases of MM were reported globally, accounting for close to 1% of all newly diagnosed cancer cases and 1.2% of all cancer-related deaths [11]. MM is often labelled as a neoplasm of ageing, with the age at diagnosis often being ≥ 65 years [9,12,13]. Very rarely, MM has been reported in individuals younger than 30 years of age at frequencies of 0.02% to 0.3% of all diagnosed cases [14]. In 1979, the Durie-Salmon staging system was developed for evaluating MM based on the presence of anemia and bone disease [15,16]. The International Myeloma Working Group defines the International and Revised International Staging systems (ISS and R-ISS) for MM based on either clinical and laboratory parameters only (ISS) or in combination with cytogenetic abnormalities (R-ISS) [17,18]. Chromosomal aneuploidy is a complex yet distinguishing feature in MM, with the disease often presenting as hyperdiploid or non-hyperdiploid, involving translocations with the IgH locus [19,20]. Fluorescent in situ hybridization (FISH) is most commonly employed to detect cytogenetic abnormalities in MM [19,20,21]. Hyperdiploidy is observed in nearly half of MM cases and is associated with a good prognosis [5,22,23]. Primary reciprocal translocations specific to B-cell chromosomal rearrangements, observed in nearly half the MGUS and MM cases, are early events in myeloma pathogenesis and are considered high-risk (HR) abnormalities as they bring oncogenes, mainly cyclins, fibroblast growth factor receptor (*FGFR*) 3, multiple myeloma SET domain (*MMSET*), and musculoaponeurotic fibrosarcoma (*c-MAF*), under the control of powerful IgH enhancers [5,22,24,25,26]. Secondary genetic alterations indicate disease progression, generally do not involve the IgH locus, and may or may not be clonal. Further, these changes are seen in a non-hyperdiploid background with reduced dependence on the BMME and often indicate a poor prognosis (Figure 3) [5,25]. Detailed classification and staging based on clinical and cytogenetic parameters are listed in Table 1.

## 2. Marrow, Microenvironment, and Multiple Myeloma

**Figure 4:**
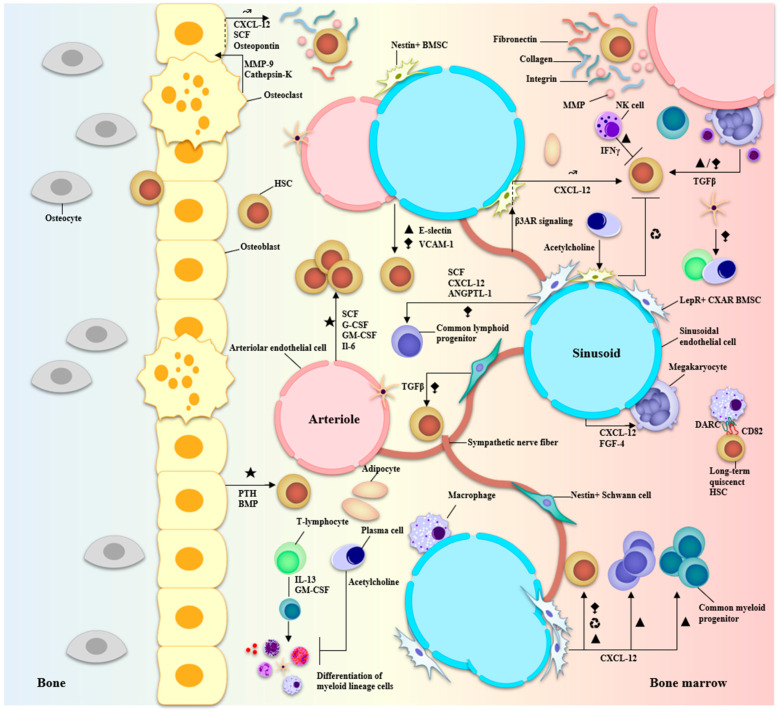
Anatomy of the BMME and niche interactions of BM-resident cells during hematopoiesis. Two major niches can be described in the bone marrow: vascular and endosteal. The vascular niche is at the core of the bone marrow comprising both arteriolar and sinusoidal vessels and is associated with LepR+ or nestin+ BMSCs and CXAR cells, macrophages, megakaryocytes, sympathetic nerve fibers, Schwann cells, endothelial cells, and adipocytes. The endosteal niche is closer to terminal bone and comprises osteolineage cells—osteoblasts and osteoclasts—involved in bone formation and recycling. Secreted growth factors, cytokines, CAMs, and ECM molecules participate in an intricate signaling network to regulate HSC quiescence and differentiation/proliferation programs. The major factors involved are CXCL-12, TGF-β, G-CSF, GM-CSF, MMPs, collagens, and integrins. Particularly, the Schwann cells and stromal cells associated with the vasculature help in the regulation of HSC migration and differentiation while cells at the endosteal surface promote HSC quiescence and maintenance of their self-renewal capacity. ★ Activation of hematopoiesis. ▲ proliferation. **⧪** retention in the BM **↝** mobilization ♻ recycling.

The BMME comprises an extracellular matrix (ECM), blood vessels, cells from the hematopoietic lineage, supporting cells required for the regulation of hematopoiesis, and cells from the osteo-lineage [39,40,41]. Normal hematopoiesis occurs in two distinct niches in the BM: the central niche which is in the core of the BM and the endosteal niche which is close to the surface of the bone and the terminal epiphysis containing the trabecular marrow (Figure 4) [40,42].

Hematopoiesis starts in the embryonic stage, primarily to produce red blood cells from transitory, non-renewing erythroid progenitor cells that carry oxygen to tissues during growth and development. This is the “primitive” phase of hematopoiesis [43,44]. As embryonic development progresses, the primitive wave allows definitive hematopoiesis to begin in various fetal niches, giving rise to erythroid-myeloid progenitor cells [43,44]. HSCs progressively move into the fetal liver and then the BM, which is the primary site for adult hematopoiesis [1,44]. The HSCs generate increasingly lineage-committed progenitor cells that give rise to all the blood cells (Figure 1). The first line of cells are the common lymphoid progenitors (CLP) and the common myeloid progenitors (CMP) [45]. Subsequent differentiation of CLP gives rise to B and T lymphocytes, which are integral for antigen-specific adaptive immunity, and to natural killer (NK) [46] cells, which are part of the innate immune system. CMPs differentiate into erythroid/platelet progenitors which produce the red blood cells (erythrocytes) and platelets (thrombocytes), and granulocyte-macrophage progenitors which produce granulocytes (neutrophils, eosinophils, basophils, and mast cells), dendritic cells (DCs), and monocytes, which differentiate into macrophages [45,47].

**Figure 5:**
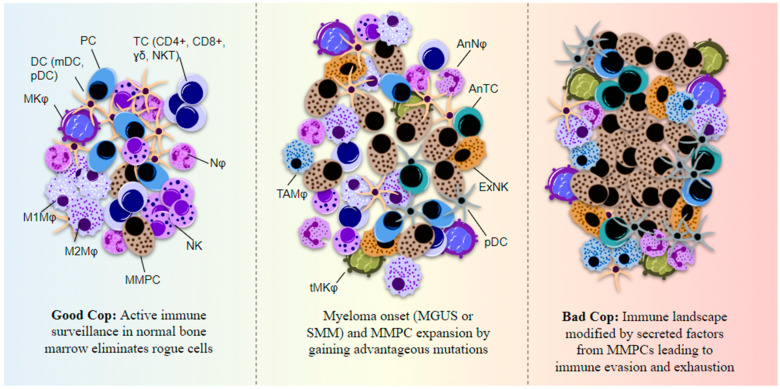
Immune cell function in myeloma BM is altered by secreted factors from the MMPCs and the cells lose their normal immune surveillance capacity. An immunosuppressive state is then established, helping MMPCs evade immune system detection. The immune cells show features of exhaustion and anergy. Myeloma cells primarily use secreted IL-6 and TGF-β to disrupt normal immune system activation and express cell adhesion molecules (CAMs) such as PSGL-1, ICAM-1, and VLA-4 which helps in attachment to various immune cells as well as the BMSCs or ECM. PD-L1 is another surface-expressed ligand on MMPCs that can bind NK- and T-cell programmed cell death protein (PD)-1 to suppress their cytotoxic functions and cause immune exhaustion. MMPCs also express CXCL-12 which results in macrophage polarization to M2 or the tumor-associated macrophage phenotype. MMPC-derived cytokines including IL-6 and IL-10, along with secreted M-CSF and VEGF, affect DC maturation and proliferation in the BM. PC = plasma cell, TC = T-cell, NKT = natural killer-like T-cell, Nφ = neutrophil, NK = natural killer cell, MMPC = multiple myeloma plasma cell, Mφ = macrophage, MKφ = megakaryocyte, TAMφ = tumor-associated macrophage, tMKφ = transformed megakaryocyte, DC = dendritic cell, mDC = monocytic DC, pDC = plasmacytoid DC, ExNK = exhausted NK, AnNφ = anergic Nφ, AnTC = anergic TC.

Disruptions to genome integrity, transcription efficiency, and cell-specific protein expression capacity affect several cellular processes including hematopoiesis [48]. Such events can drive the malignant transformation of BM cells and alter the environment in a manner disruptive to normal cells but favorable for their own survival and proliferation [49]. A double-edged hypothesis is suggested by several studies whereby both the microenvironment and the hematopoietic cells themselves are suggested to initiate or promote malignancy [42,50,51,52]. Cells of the hematological lineage and the BMME can co-evolve to promote tumorigenesis, cancer progression, and the development of drug resistance. In MM, the BMME exhibits distinct, stage-specific profiles (Figure 5).

### 2.1. ECM Components

The ECM proteins in the BMME are fibronectin, collagens, laminin, tenascin, thrombospondin, and elastin. The ECM also contains other glycoproteins, cell adhesion molecules (CAMs), proteases, and growth factors required for tissue scaffolding and homeostasis [53,54,55,56,57]. Glycoproteins and collagens help in the adhesion and expansion of HSCs and support the development, proliferation, and maturation of B lymphocytes and erythroid lineage cells. Fibronectin provides anchorage for hematopoietic progenitor cells and plays a crucial role in myelopoiesis and thrombopoiesis [46,58,59]. Proteoglycans, particularly heparan sulphate, are important for differentiation, lineage specification, and homing during hematopoiesis [60,61,62,63]. CAMs and integrins are essential for homing and regulating the survival and proliferation of HSCs [56,64,65]. Serine proteases such as CD26, neutrophil serine proteases, and matrix metalloproteases (MMPs), especially MMP-9, are required for the mobilization of HSCs during hematopoiesis by cleavage of interactions with specific adhesion molecules such as integrins, vascular CAMs (VCAMs), and fibronectin, and also in association with growth factors like granulocyte colony-stimulating factor (G-CSF) and granulocyte-monocyte colony-stimulating growth factor (GM-CSF) [66,67].

Transcriptomic analysis of MM cells showed enrichment for genes associated with the ECM [68]. Dysregulation of fibronectin, collagens, and lysyl oxidase (LOX) leads to BM fibrosis and is a poor prognostic factor and indicator of disease progression in hematological malignancies, particularly MM [68,69,70,71]. Fibronectin binding by MM cells triggers the activation of nuclear factor (NF)-κB signaling and the development of cell adhesion-mediated drug resistance (CAMDR) [23,69]. Collagen expression varies with disease stage, with the BM of MM patients having high plasmacytosis showing lower levels of collagen I but increased collagen IV compared to MGUS and normal BM [69]. As the disease progresses, collagens I, II, and III are re-expressed, indicating supportive roles in aggressive disease [68]. MMP-2 and MMP-9 are known to be overexpressed in MM and cause lytic bone lesions by degrading collagens I and IV [72,73]. Drug-resistant phenotypes were observed with MM patient BM cells cultured on fibronectin or BM stromal cells (BMSCs), alongside increased expression of CAMs in those with post-chemotherapeutic drug resistance. Further, tumor necrosis factor (TNF)-mediated apoptosis resistance was also observed from the cells cultured with BMSCs but not fibronectin [69]. Serglycin is an intracellular proteoglycan exclusively found in immature myeloid cells in the BM and is found to be upregulated in MMPCs where it could potentially create a pro-inflammatory environment and alter bone development [74]. Increased levels of laminin and laminin receptors mediate progression from MGUS to MM and promote migration [75].

### 2.2. Vascular Compartment

The vascular niche of the BM consists of a network of blood vessels arising from BM-resident endothelial cells (BMECs) and perivascular stromal cells, which play critical roles in cellular trafficking, hematopoiesis, and osteogenesis [76,77,78,79,80]. Hematopoiesis in the BM also depends on blood vessel permeability [77].

#### 2.2.1. Vasculature-Associated Cells

Maintenance of adult HSCs is mediated through surface expression of CAMs like C-X-C motif chemokine ligand (CXCL)-12, nestin, and melanoma CAM (MCAM) (CD146), expressed differentially by BMECs and BMSCs [77,78,80,81,82,83]. Cytokines and CAMs secreted by different subsets of BMECs are important for the regulation of homing and the differentiation of HSCs. E-selectin helps in the homing of HSCs to the BM and proliferation, while VCAM-1 is essential for HSC retention [76,79,84]. Platelet endothelial CAM-1 (PECAM-1) (CD31)-expressing endothelial cells are found in the BM across all developmental stages and are also expressed on the surface of both myeloid and pre-B-lymphoid hematopoietic progenitor cell subsets [85]. In mice, CD31+ microvascular endothelial cells have been shown to rescue short- and long-term multilineage hematopoiesis following lethal irradiation of the BM [86]. Endothelial cell-secreted cytokines have both pro- and anti-hematopoietic activity. Stem cell factor (SCF), GM-CSF, interleukin (IL)-6, IL-1α, IL-11, and G-CSF are reported to stimulate hematopoiesis while thymosin-β4 is an inhibitor [87,88]. BMEC-secreted chemokines such as CXCL-12 and fibroblast growth factor (FGF)-4 are important for the localization of megakaryocyte progenitors to the vascular niche for the production, maturation, and release of platelets through VCAM-1 and VLA-4 signaling [89,90]. Granulopoiesis and lymphopoiesis are supported by BMSCs [80,91]. Leptin receptor (LepR)+ BMSCs, including skeletal stem cells, are rare during early development but are more abundant in adulthood and produce high levels of SCF, CXCL-12, and angiopoietin-like 1 (ANGPTL-1), which are required for HSC maintenance [80,92]. A subset of lymphoid progenitors is found in the periarteriolar niche and depend on secreted SCF from LepR + osteolectin+ BMSCs for their maintenance in this niche. Another subset of LepR+ BMSCs also secretes IL-7 for early lymphoid progenitors. However, the exact niche occupied by them is uncertain [80,92].

BMECs are “activated” in MM conditions, contributing to angiogenesis, immune escape, ECM adhesion, migration, proliferation, and entry of myeloma cells into circulation [93]. Secretion of cytokines and growth factors like vascular endothelial growth factor (VEGF), IL-6, and insulin-like growth factor (IGF)-1 by BMECs support MM cell growth in the BMME [94]. CAMs such as homing CAM (HCAM), very late-activation antigen (VLA)-4, intracellular CAM (ICAM), and neural CAM (NCAM), produced by MMPCs and their regulatory partners like integrins, cadherins, and IgCAMs, are critical for progression from precursor conditions to active myeloma [95,96,97]. Although CAMs appear to be required for PC homing to the BM, expression of NCAM and lymphocyte function-associated antigen (LFA)-3 was notably higher in MMPCs while selectin-1 and VLA-2 were absent [95]. CXCL-12 produced by BMSCs is important for neovascularization and ECM adhesion of MM cells, invasion, and chemoresistance [98,99]. VEGF is important in BMEC differentiation and crucial for MMPC migration, with increased levels detected in aggressive MM plasma compared to earlier stages and correlated to higher levels of circulating endothelial progenitors, indicating a role in BMEC recruitment for neovascularization [100,101,102]. VEGF receptor 2 (VEGFR2) has been targeted in a murine MM model and shown to inhibit angiogenesis and tumor growth [103]. E-selectin is essential for homing and maintaining MMPCs in the supportive BMME and evading bortezomib cytotoxicity, with higher levels observed in advanced-stage and drug-refractory MM [95]. MMPCs attach to BMSC fibronectin and collagen via ligands such as VLA-4 and syndecan-1, respectively, and attachment with BMECs is facilitated by CD44 interaction with endothelial hyaluronic acid [95].

#### 2.2.2. The Blood Vessel Niches

Two distinct types of blood vessels can be identified in the BM: arterial (CD31 high/CD45−/Sca-1 high/Nestin+) and sinusoidal (CD31+/CD45−/Sca-1 low/Nestin−) [77]. Highly quiescent HSCs are found close to the endosteal surface and away from regions with rapid blood flow [104]. Nestin+ non-myelinating Schwann cells have been found in the arteriolar niche, where they help to maintain hibernating HSCs through activation of the transforming growth factor (TGF)-β pathway [76,77,105]. Further, Nestin+ cells in arterial blood vessels co-localize with HSCs and serve as a site for leukocyte trafficking and homing to the BM [76,77,81]. Knockout studies have shown that multipotent Nestin, CXCL-12, SCF, and LepR+ stromal cells, which localize near the sinusoidal and arteriolar endothelium, are essential for supporting hematopoietic cell maintenance and differentiation [106,107,108]. Sinusoidal megakaryocytes appear to have dual roles in regulating HSCs: maintenance of HSC quiescence via CXCL-4 and TGF-β [109,110] while supporting proliferation of HSCs via FGF-1 [109,111].

In hematological malignancies, tumor cells have been shown to compete with HSCs for niche occupancy in the early stages and eventually evolve niche independence with progression [112,113]. Like normal PCs, MMPCs also employ CXCR-4-CXCL-12 signaling to migrate via sinusoidal vessels to the BM [114]. P-selectin glycoprotein ligand 1 (PSGL1) expressed on MMPCs interacts with endothelial P-selectin and facilitates the “rolling” of MM cells in BM microvasculature [114]. It has been hypothesized that reduced CXCR-4 expression on MMPCs, which is also shown to occur with bortezomib treatment, promotes their egress from the BM to peripheral blood to establish extramedullary disease by detaching MMPCs from BMSCs [115].

### 2.3. Osteolineage Cells

Mesenchymal stem cells produce the osteolineage cells—chondrocytes, adipocytes, and osteoblasts—which have important roles in hematopoiesis [105,116,117,118]. One of the major complications in MM is lytic bone disease which occurs due to imbalances in osteolineage cells, with hyperactive osteoclasts and decreased osteoblast activity.

#### 2.3.1. Osteoblasts

Endosteal osteoblasts are known to be an HSC niche [116,118,119]. Osteoblasts promote adult HSC proliferation and maturation via parathyroid hormone (PTH) and bone morphogenic protein (BMP) signaling [120,121,122]. Osteoblast-secreted cathepsin-X degrades CXCL-12, which is required for HSC engraftment, homing, maintenance, and mobilization in the BM niches [123]. In mice, depletion of osteoblasts caused niche migration of HSCs from the BM to the spleen and liver and showed impairment in B lymphopoiesis [124,125]. However, emerging evidence indicates that mature N-cadherin-expressing osteoblasts are not essential for maintaining adult hematopoiesis in the BM [126,127,128]. CD146+ stromal osteoprogenitor cells produce angiopoietin-1 (ANGPT-1), which is important for maintaining the vascular HSC niche [129].

MM cells residing in the osteoblastic niche are protected from apoptosis, have more oncogenic potential, and inhibit osteoblastogenesis [130]. Stimulation of osteoblasts with PTH-1 or bortezomib has been shown to block cell growth in MM model systems [131,132]. Osteoblasts secrete decorin-A to induce cell cycle arrest and apoptosis of myeloma cells and recruit immune cells to the BM for potential anti-tumor activity. In contrast, inhibition of osteoblast growth by myeloma cells is achieved through DKK-1 signaling [133]. Cell-to-cell contact between VLA-4 on myeloma cells and VCAM-1 on osteoblast progenitors suppresses Runt-related transcription factor 2 (RUNX2)/core binding factor-α1 (CBFA1)-mediated transcription and inhibits osteoblast formation [134,135].

#### 2.3.2. Osteoclasts

Osteoclasts are bone-chewing cells that secrete enzymes such as MMP-9 and cathepsin K, which degrade CXCL-12, SCF, and osteopontin on the osteoblast surface and promote HSC mobilization [116,136]. Osteoclasts play an indirect role in B-lymphocyte development by mobilizing their progenitors to BM niches via expression of CXCL-12 and IL-7 in stromal cells [137]. Another indirect mechanism of hematopoietic regulation by osteoclasts is by modulating osteoblast differentiation [138]. It has been suggested that osteoclasts form an HSC niche, based on observations from a mouse model where mice depleted in osteoclasts developed osteopetrosis that caused extramedullary hematopoiesis [138,139,140].

It is well known that osteolytic activity is abnormally increased in MM leading to lytic bone disease, fractures, and release of growth factors outside the bone, thereby promoting aggressive disease [133,141,142]. VLA-4–VCAM-1 contacts are essential not only in the suppression of osteoblastogenesis but also for increased osteoclast activity [134]. MM cells cause upregulation of receptor activator of NF-κB (RANKL) and downregulation of osteoprotegerin (OPG) secretion by BM-MSCs (via VLA-4 interactions) and osteoblasts, leading to osteoclast differentiation and hyperactivity [143,144]. Other cytokines and factors overexpressed in the MM BM such as macrophage inflammatory protein (MIP)-1α and IL-3 promote osteoclastogenesis and inhibit osteoblast formation in a stage-dependent manner [141]. Proteasome inhibitors (PIs) are also helpful in targeting osteoclasts via inhibition of nuclear factor (NF)-κB and P38/MAPK signaling and pro-inflammatory cytokines such as MIP-1α, IL-6, TNF-α, and IL-1β [145,146].

#### 2.3.3. CXCL-12-Abundant Reticular (CXAR) Cells

Recently, it has been demonstrated that mesenchymal CXAR cells—progenitors of the adipo-osteogenic lineage of cells—are essential for releasing HSCs from quiescence as well as for maintaining them in an undifferentiated state. CXAR cells also play a role in HSC self-renewal and proliferation of lymphoid and myeloid progenitors [147,148,149]. Most perivascular HSCs are in direct contact with CXAR cells and Nestin+ stromal cells in this niche [150]. Further, HSPCs and lymphoid progenitors in the BM are found in close association with CXAR cells in the BM [148,151]. Studies have demonstrated the importance of CXAR-cell chemokine signaling for HSC maintenance in the BM. Greenbaum et al. have shown that depletion of CXCL-12 from CXAR cells causes mobilization of HSPCs from the BM and B-lymphoid progenitor loss [152]. In mice, treatment with LPS increased CCL-2 (macrophage chemoattractant protein (MCP)-1) expression in CXAR cells and other BM MSCs required for monocyte egress into the bloodstream [153].

CXCL-12 is important for MM cell evasion of apoptosis and survival by upregulating survivin, B-cell lymphoma (BCL)-2, and ATP-binding cassette (ABCC)-1, a multidrug resistance transporter [154]. Further, CXCL-12 promotes homing of CXCR-4-expressing MM cells to CXCL-12+ stromal niches in the BM and retention through strong adhesion of α4β1 integrin on MMPCs to VCAM-1 in the BM microvasculature [114,155].

#### 2.3.4. Adipocytes

In the adult BM, adipocytes are reported to negatively regulate hematopoiesis, although emerging evidence indicates their contribution to hematopoiesis through lineage-specific differentiation [156,157,158,159]. One factor attributed to lesser hematopoietic support by BM adipocytes is the reduced production of growth factors such as G-CSF and GM-CSF [156,160]. Characterization with electron microscopy revealed that mouse BM adipocytes are innervated by sympathetic nerves and found close to endothelial cells in the sinusoidal vasculature. This study also identified BM adipocytes interacting with developing myeloid/granulocyte lineage cells and erythroblast islands [161]. Differential numbers of CD45+ HSCs have been reported in mice with adipocyte-rich tail BM having two- to three-fold lower numbers compared to adipocyte-poor thoracic BM [156]. Human BM adipocytes were shown to support the complete differentiation of CD34+ HSPCs into myeloid and B- and NK-lymphoid lineages but did not help maintain immature HSC quiescence in a co-culture model [162].

BM adipocytes in MM show altered expression profiles with decreased adiponectin and increased inflammatory cytokines, adipokines, and free fatty acids, which induce lipolysis of adipocytes and support MM cell proliferation and metastasis [163]. These altered BM adipocytes in MM also promote aggressive bone disease [164]. Low serum adiponectin levels have been reported in MGUS and MM patients, indicating a myeloma-suppressive role [164,165]. Adipocyte-derived MCP-1 and CXCL-12α are important for the recruitment of MMPCs to the BM where BMME cell-secreted growth factors like IL-6, TNF-α, IGF-1, and hepatocyte growth factor (HGF) promote their retention, proliferation, and survival by evading apoptosis and inducing chemoresistance [166]. In a mouse model, direct cell-cell adhesion with adipocytes increased DNA synthesis and prevented apoptosis of MM cells [167]. The association of increased leptin levels has been noted with increasing MM stage and could potentially have a role in the progression from MGUS to active MM through OPG/RANKL signaling [24,165]. These data suggest that BM adipocytes support myeloma progression.

### 2.4. Supporting Cells

Supporting cells are also involved in the direct or indirect regulation of hematopoiesis. Neurons of the sympathetic nervous system (SNS) influence osteoblast activity and the osteocyte network and mediate hematopoietic progenitor cell mobility through G-CSF [168]. Further, sympathetic nerve fibers use β3 adrenergic receptor signaling to regulate CXCL-12 expression in Nes+ mesenchymal and stromal progenitor cells, which is required for HSC mobilization from the BM [81,169]. Adrenergic signaling mediated by the interaction of CCR7 and CXCR-4 with the β2 adrenergic receptor and concerted chemokine expression is also important for leukocyte release from the lymph nodes as well as their homing to the BM through endothelial adhesion molecule signaling [169,170,171].

The SNS is a well-known driver of malignancy by modulating the BMME in acute lymphoblastic leukemia (ALL) [172]. However, reduced adrenergic signaling leading to sympathetic neuropathy supports myeloid malignancies, as shown in mice studies [173,174]. In fact, β-adrenergic receptor blockers have been used with some success in MM patients to reduce overall mortality and in MM cell lines to prevent proliferation and survival [175,176].

### 2.5. Hematopoietic Progeny

Lineage-specific cells such as megakaryocytes, macrophages, neutrophils, and T lymphocytes arising from the differentiation of HSCs contribute to the regulation of hematopoiesis by manipulating HSC quiescence and mobilization across niches. Further, these cells themselves influence and are influenced by malignant PCs during the development and progression of myeloma (Figure 3).

#### 2.5.1. Megakaryocytes

Megakaryocytes promote HSC proliferation and quiescence through TGF-β1 and CXCL-4 signaling and, under stress, secrete FGF and IGF-1 to activate HSC proliferation [109,110,177]. Further, it has been shown that megakaryocytes are closely associated with platelet- and myeloid-biased HSCs [177,178]. The myeloid-biased HSCs express megakaryocyte-like transcripts such as transcription factor GATA1 (GATA-binding protein 1), the TPO (thrombopoietin) receptor Mpl, and the platelet and endothelial aggregation factor VWF (von Willebrand Factor), as well as the megakaryocyte-specific integrin CD41 [179]. It has also been shown that the depletion of megakaryocytes leads to decreased thrombopoietin (TPO) expression, resulting in an overall reduction in HSC numbers in the BM [180]. Further, megakaryocyte ablation has also been shown to reprogram the associated VWF+ myeloid-biased HSCs towards more lineage-balanced HSCs [178]. Recently, a feedback mechanism has also been proposed whereby microparticles released by megakaryocytes can elicit differentiation of HSCs and HSPCs into functional megakaryocytes [181].

The specific role of megakaryocytes in myelomagenesis still needs to be studied. However, it has been reported that cytokines such as TPO, IL-6, and soluble P-selectin, which participate in myeloma progression, are also required for thrombopoiesis and megakaryocytopoiesis [182]. In a study by Lemancewicz et al., newly diagnosed MM (NDMM) patients had significantly increased levels of cytokines, including TPO and soluble P-selectin, compared to controls. Further, stage-specific differences in cytokine expression and megakaryocyte numbers were observed [182]. In mouse cell lines, megakaryocytes were shown to be necessary for MMPC growth in the BM, and this could be through IL-6 and APRIL [183,184]. IL-1β is produced by myeloid and megakaryocytic cells and myeloma cells and could induce osteoclast activity, mediate homing and adhesion of MMPCs in the BM, and consequently induce pro-inflammatory IL-6 signaling [185].

#### 2.5.2. Macrophages

Resident macrophages in the BM contribute to HSPC retention and use G-CSF for their egress into circulation [169,186,187]. It has been reported that CD234 (Duffy antigen receptor for chemokines (DARC))-expressing macrophages mediate long-term HSC quiescence by binding CD82/KAI1 and downstream TGF-β1/Smad3 signaling [188]. Resident macrophages indirectly regulate HSC maintenance through interactions with osteoblasts and Nestin+ MSCs [189]. Through depletion experiments, CD169+ VCAM-1+ macrophages were shown to be essential for erythropoiesis and HSPC and progenitor retention in the BM [190]. Macrophages expressing α-smooth muscle actin also indirectly regulate hematopoiesis via prostaglandin E2, which enhances CXCL-12 production by Nestin+ cells [191,192].

In MGUS and MM patient BM, macrophages were closely associated with MMPCs, with high levels of CD68+ macrophages, a indicator of aggressive disease and poor prognosis [193,194,195,196]. Interaction of MM cells with BMSCs via CXCL-12 results in the recruitment of monocytes from peripheral blood, specifically macrophages, and polarizes them towards M2 or a tumor-supportive phenotype and prevents chemotherapy-mediated MM cell apoptosis [197,198]. Macrophages are important for MMPC proliferation, migration, homing to BM, survival, development of drug resistance, immunosuppression, and angiogenesis [199,200,201,202,203,204,205]. M2 macrophages, also known as tumor-associated macrophages (TAM), produce IL-6 and other cytokines (IL-10, IL-12, VEGF, IL-1β, IGF-1) important for MMPC proliferation [200,206,207]. MMPCs expressing PSGL1 and ICAM-1 can polarize macrophages towards the M2 or TAM phenotype in co-culture models by enhancing CD206 expression and also induce drug resistance through the Src, ERK, and c-MYC pathways [205]. MMPCs secrete pro-angiogenic VEGF and FGF-2 and convert macrophages to an endothelial cell-like phenotype to produce abnormal vasculature in a “vascular mimicry” process [208]. IL-10 and TGF-β secreted by TAMs, along with IL-32 secreted by MM cells, are thought to play a role in immune suppression by reducing T-cell surveillance through reduced MHC-II activation and decreased production of IFN-γ, IL-2, and TNF-α [197,198,209,210].

#### 2.5.3. Neutrophils

During acute infection or inflammation, neutrophils promote emergency myelopoiesis by reactive oxygen species (ROS) production through HSPC proliferation. Post-transplantation and upon sinusoidal injury, a subset of Gr1+CD115− neutrophils secrete TNFα to drive sinusoidal regeneration and endothelial cell proliferation, which are niche components vital for normal hematopoiesis [211,212]. CD62L-low CXCR-4-high neutrophils are identified as “aged” and undergo circadian clearance from the BM, consequently causing a size reduction of the hematopoietic niche [213]. Interestingly, neutrophils in the intestine also have a paracrine effect on granulopoiesis in the BM by suppressing IL23-mediated signaling [214].

Neutrophils are implicated in immune evasion and chemoresistance development in MM [215,216,217,218]. The gene expression profile of MM patient peripheral blood neutrophils was found to be different from that of healthy normal neutrophil gene expression, particularly in FC-γ-R-mediated phagocytosis, endocytosis, leukocyte transendothelial migration, chemokine signaling, and TLR pathways, with little evidence for their role in T-cell suppression [219]. However, some genes such as CSK, GSA, MEGF, PGM1, and PROK2 were found to be upregulated in high-density neutrophils from the BM of MM patients and were associated with progression from MGUS to MM, and mature neutrophils alone were shown to impact MM patient survival [114].

#### 2.5.4. T Cells

All sub-populations of T cells including CD4+ T-helper cells, CD4+ regulatory T cells, CD8+ T cells, γδ-T cells, and NK-T cells produce cytokines important for regulating hematopoiesis [220,221]. Th1 cells help maintain the homeostasis of hematopoietic progenitor cells (HPC). Reduced Th1 activity coupled with low Stat4 expression and increased Th2 cells results in reduced HPC numbers and proliferation in the BM, while increased Th1 activity with reduced Stat6 expression is favorable for HPC proliferation [220,222,223]. In mice, CD4+ Th cells were shown to be important for the development of myeloid lineage cells from their progenitors [223]. Activated Th1 cells are important for the differentiation of macrophages through secreted IL-3, GM-CSF, and M-CSF. However, secretion of IFNɣ and TNFɑ have the contrasting effect of suppressing hematopoiesis. Th2 cells also produce IL-3 and GM-CSF similar to Th1 cells while producing lineage-specific cytokines such as IL-4, IL-5, IL-9, and IL-13, which influence hematopoiesis [220]. IL-5 produced by T cells is required for eosiniophilopoiesis both during primitive hematopoiesis and during allergen sensitization at later life stages [224,225]. Another important T-cell subset, Th-17 cells, produces IL-17, which is important for neutrophil homeostasis by controlling tissue migration and neutrophil apoptosis [226].

T-cells are known to be altered in numbers and function in MM. There is a reduction in the overall CD4+ and CD8+ T-cell population, skewed Th1/Th2 ratio, and T-cell anergy [227,228]. Nearly half of the T-cell population described in MM are of the CD3 + CD8 + CD57+ effector-memory subtype and lack surface TCR expression [228,229]. Flow cytometric analysis revealed increased expression of programmed cell death protein (PD)-1, cytotoxic T lymphocyte-associated protein (CTLA)-4, CD160, and 2B4 (CD244/signaling lymphocytic activation molecule (SLAM) F4)) in the BM T-cells of MM patients, indicative of an immunosuppressive microenvironment favorable for immune evasion by MM cells [230]. MMPCs express the ligand PDL-1 for the T-cell PD-1 receptor, and this interaction results in immune suppression due to impaired T-cell proliferation and cytokine secretion with T-cell exhaustion, promoting relapse of MM in a mouse model [227,231]. This also correlates with clinical studies showing increased PD-1+ T-cells in the BM of relapsed/refractory (RR)MM compared to NDMM or MM-under-remission patients [227]. MM cells also secrete TGF-β, which reduces IL-2-mediated T-cell proliferation [231,232]. By “trogocytosis”, T-cells acquire MM cell surface-derived proteins upon contact and establish a novel subset of regulatory T-cells, often without functional significance, but potentially serving as prognostic markers or therapeutic targets [233]. Other MM-specific T-cells are CD57+ CD8+ terminal effector T-cells detected from both BM and peripheral blood, but their function is unclear [234]. Th17 cells have also been reported from both the peripheral blood and BM of MM patients. Whereas IL-17 promotes tumor growth, Th17 cells are correlated with long-term survival in MM patients [228]. A small subset of γδ-T cells was also identified with adaptive and innate immune responses against myeloma [235].

#### 2.5.5. B Cells

BM B lymphocytes also play a role in regulating normal hematopoiesis. Patel and Pietras have demonstrated that acetylcholine secreted by B cells during both homeostasis and inflammation acts on Chrna7+ mesenchymal stromal cells and, in turn, promotes expression of CXCL-12 and ANGPT1 in the HSC niche, leading to arrest of HSC cell cycling and myeloid lineage differentiation [236]. CD138+ BMPCs are crucial for myelopoiesis in the murine BMME. A subset of “myeloid-like B cells” promotes emergency myelopoiesis during systemic infection, primarily through IL-10 [237,238]. In mice, a subset of macrophages has also been observed to originate from pre/pro-B-cells expressing non-rearranged BCR genes and co-expressing both myeloid and lymphoid lineage markers [239].

MM is a B-cell malignancy, and MMPCs would significantly influence and be influenced by the BMME. MMPCs establish crosstalk with almost all the BM’s cell types, ECM, and vasculature to harness them for their proliferation and survival [8]. MMPCs are late-stage B-cells expressing the characteristic markers CD138 (syndecan-1), CD38, and monoclonal Ig light-chain, but lacking the CD19 marker for pre-differentiated B-cells and other B-cell lymphomas. In addition, they do not frequently express other B-cell antigens such as CD20, CD22, CD24, and CD45 [240]. MMPC VLA-4 binds to VCAM-1 on BMSCs while MMPC CD138 and VLA-4 bind to the BM-ECM collagen-1 and fibronectin, respectively, and in turn help in BM adhesion of MMPCs and inducing IL-6 transcription [241,242]. The major cytokines secreted by MMPCs and involved in myeloma cell proliferation, survival, drug resistance, migration, angiogenesis, and osteolysis are IL-6, TNF-α, VEGF, and IGF-1 [243]. Older studies have reported circulating pre-B cells and clonotypic and possibly non-malignant PCs from the peripheral blood of MM patients. However, the latter could be indicative of MGUS [244,245,246]. MMPCs have been shown to bind soluble IFN-γ1, supporting MMPC proliferation and dexamethasone resistance [247]. Clonogenic populations of PC progenitors have also been identified from the BM of MM patients, some of which are CD19+, confer drug resistance, and contribute to MRD as potential stem cells for MM recurrence [248,249].

#### 2.5.6. NK Cells

NK cells appear to have contradictory roles in regulating hematopoiesis as they produce both growth factors and inhibitory cytokines [250,251]. In vitro cell culture and murine in vivo studies have shown that IL-2-activated NK cells promote hematopoiesis, particularly affecting megakaryocyte and granulocyte lineages in the absence of other cytokines or growth factors. Conversely, IL-2-activated NK cells have been shown to have an inhibitory effect on hematopoiesis in the presence of cytokines and other normal growth conditions, partly via IFN-γ signaling [250].

Conflicting reports exist regarding NK cell numbers in MM, but it is known that NK-cell-mediated cytotoxicity is reduced and worsens with the advancement of MM [252,253,254,255,256]. Cytokines such as TGF-β, IL-10, IL-6, and prostaglandin-E2 (PGE2) secreted by MM cells promote MM cell proliferation and suppress NK cell activity through downstream signaling via STAT-3, which inhibits IFN-γ-mediated antibody-dependent cellular cytotoxicity (ADCC) and reduces the NK cell response to inflammatory cytokines such as IL-12 and IL-15 [256]. Further, the monoclonal Igs secreted by myeloma cells affected NK cells, as shown in a study where the use of monoclonal IgA on NK cells resulted in a reduction of cytolytic granules and the creation of vacuoles [257]. In an MM mouse model, the BM effector Killer Cell Lectin-Like Receptor (KLR)G1-NK cell subpopulation was substantially reduced with myeloma progression due to impaired chemokine receptor-ligand ratios with downregulation of CXCR-3 and CXCR-12 and increased CXCL-9 and CXCL-10 [258]. NK cells from MM patients show a reduction in activating receptors NKG2D (aka killer cell lectin-like receptor K1/KLRK1), DNAX accessory molecule (DNAM)-1, and CD161, and an increase in inhibitory CD158a (inhibitory Killer Ig-like Receptor/KIR). NKT cells that express both NK- and T-cell receptors also show impaired cytotoxicity [114]. MM cells also express the PD-L1 ligand that can bind PD-1 on T and NK cells and suppress DNAM-1 on these cells to prevent activation of immune responses [256].

#### 2.5.7. Dendritic Cells (DCs)

BM-resident DCs retain recirculated mature B- and T-memory cells [259,260]. In mice, perivascular DCs formed clusters that were embedded with recirculating B- and T-cells, and selective ablation of these DCs resulted in the elimination of mature B-cells from this niche [259]. Monocytes, including DCs, secrete G-CSF, which is important for HSPC mobilization [261]. Bone-marrow-resident DCs found close to venous sinusoids and arterioles use CXCR-2 signaling to regulate endothelial function and are important for the mobilization of hematopoietic stem and progenitor cells (HSPC) [262]. It is also reported that classical DCs expressing CD8α (murine) or X-C motif chemokine receptor 1 (XCR1) and C-type lectin domain containing 9 (CLEC9) (human) induce erythropoiesis during stress [263].

DC dysfunction is well-studied in MM, with plasmacytoid DCs (pDC) particularly supporting myeloma cell proliferation, survival, and immune evasion [264,265,266,267]. DCs from MM patients have been shown to lack surface markers such as CD80 and CD86, and have a higher prevalence of immature and inactive DC types [268,269]. MMPC-derived IL-6, IL-10, M-CSF, and VEGF inhibit DC maturation, development, and antigen presentation capacity by driving DC progenitors toward non-specific or inactive monocytic lineages [269]. DCs in MM can promote myeloma cell proliferation and produce IL-17 to expand the BM Th-17 cell population, which then plays a role in osteoclastogenesis and bone lysis [270]. Thus, DC dysfunction significantly drives MM’s immune evasion and drug resistance.

### 2.6. Clonal Hematopoiesis and Development of Multiple Myeloma

A subset of HSCs in the BM acquires advantageous somatic mutations over time and undergo selective expansion in a process termed “clonal hematopoiesis” (CH) [271,272,273]. When some of these mutations are oncogenic, it is termed clonal hematopoiesis of indeterminate potential (CHIP), which leads to establishing precursor conditions like MGUS and malignant transformation to active MM [271,272]. CHIP is rarely detected in the younger population but occurs at a frequency of up to 10% in those over 70 years [273]. Frequently mutated genes in CH are the epigenetic regulators Tet Methylcytosine Dioxygenase 2 (*TET2),* DNA methyltransferase *(DNMT3A)*, and additional sex combs like 1 *(ASXL1)* [273,274]. CHIP-associated variants are indicators of high-risk disease with poor prognosis and response to treatment in MM, particularly in those undergoing transplant [275,276]. Identification of CHIP is based on the presence of at least 2% higher variant allele frequency (VAF) in genes associated with hematological malignancies as determined from sequencing data with no indications of hematopoietic dysplasia or abnormal blast counts [273,277].

## 3. Immune Profiling Using Single-Cell Transcriptome Sequencing in Multiple Myeloma

In hematological malignancies, immune system dysfunction is well-established, and a deeper understanding of the immune landscape can inform precision diagnostics, drug response prediction, and targeted treatment. Recent technological advancement has seen an influx of several non- and minimally invasive approaches for individualized immune profiling from limited samples to obtain gene/protein expression information from bulk immune cells and single cells [278]. In this section, we attempt to review the application of one of the most promising technologies in this area—single-cell transcriptome sequencing (SCTS)—in immune profiling of hematological malignancies, focusing on MM. Table 2 provides a detailed list of recent single-cell sequencing studies in MM focusing on the immune microenvironment.

SCTS is an emerging technology used to resolve gene expression differences between cell populations of a heterogeneous tumor sample, and it has been employed in hematological malignancies to understand aberrant immune cell frequency and function. Many SCTS studies have been carried out for MM in recent years, focusing on the BM tumor microenvironment, the immune landscape and immune evasion, and the development of drug resistance and relapse.

Single-cell sequencing of around 75,000 cells from 14 MM patients by Liu et al. provided insights into the malleability of the tumor microenvironment and its role in tumor heterogeneity and MM progression, as demonstrated by co-clustering patient immune and PCs with similar genetic backgrounds [282]. The authors also traced the journey of PCs from SMM to active myeloma and relapse using somatic alteration, cell type-specific marker expression, and gene expression data in relation to the tumor microenvironment. Unlike the malignant cells, the clustering of non-malignant cells was independent of tumor stage or origin. However, patient-specific differences in expression were observed, further underscoring the involvement of the tumor microenvironment’s interaction with the genetic background [282]. BM cells from different stages of MM progression and healthy donors were used for SCTS, revealing that NK cell abundance increases early in the disease along with altered chemokine receptor expression. In this study, the authors found that granzyme K+ cytotoxic T-memory cells were lost as early as SMM and could play a critical role in MM immunosurveillance in mouse models. Additionally, major histocompatibility complex (MHC)-class II dysregulation in CD14+ monocytes leads to T-cell suppression in vitro [281]. A compromised tumor microenvironment was also demonstrated in a study combining SCTS with validation using bulk RNA sequencing (RNAseq), flow cytometry, and functional experiments [285]. Results showed enrichment of exhausted NK cells and CD8+ T cells, macrophage reprogramming to a mixed M1-M2 phenotype, and two TAM clusters present only in the MM stage with increased M2 scores. Interaction analysis showed enrichment for two ligand–receptor pairs between macrophages and malignant PCs involving phagocytosis suppression and the macrophage inhibitory factor, which alters macrophage phenotype, and these alterations correlated with disease progression and adverse outcomes. In a multi-omics study that included SCTS as part of the MMRF immune atlas pilot project, 18 MM patient BM samples were used to compare immune cell profiles using scRNA-seq, cytometry by time-of-flight (CyTOF), and cellular indexing of transcriptomes and epitopes by sequencing (CITE-seq). The techniques exhibited differences in identifying T-cell, macrophage, and monocyte numbers. Overall, ISS stage 3 patients had decreased CD4+ T/CD8+ T-cell ratios, and ras-related C3 botulinum toxin substrate 2 *(RAC2)* and proteasome 20S subunit beta 9 *(PSMB9)* expression was upregulated in NK cells of progressive versus non-progressive MM [283]. SCTS of 10 MM individuals pre and post two cycles of a bortezomib-cyclophosphamide-dexamethasone (VCD) regimen revealed increased immune-reactive and stress-associated pathways while unfolded-protein response (UPR) and metabolic-related programs were reduced. Low immune-reactive gene expression indicated poor survival and non-responsiveness to drugs, likely by reduced MHC-class I-mediated antigen presentation capacity (APC) and immune surveillance, and upregulation of immune escape genes. The authors also found a connection between the tumor-intrinsic immune reactive program and the immunosuppressive microenvironment arising from immune cell exhaustion and checkpoint molecule expression in T-cells, NK cells, and monocytes [284]. Another study focusing specifically on SCTS of DCs and monocytes from 10 MM patients found five distinct clusters for each cell type [286]. Using trajectory analysis, one subset—monocyte-derived DCs (mono-DCs)—was shown to be generated from intermediate monocytes. Compared to healthy controls, conventional DC2 (cDC2), mono-DC, and intermediate monocytes of MM patients exhibited impaired APC. Additionally, the regulatory function of interferon regulatory factor 1 (IRF1) was shown to be decreased in the cDC2, mono-DC, and intermediate monocytes of MM patients.

## 4. Immunotherapy in Multiple Myeloma

Immunotherapy has come a long way from the Nobel Prize-winning first documented use of allogeneic bone marrow hematopoietic cell transplantation in 1968 by Edward Donnall Thomas for leukemia treatment [287]. The primary immunotherapies for hematological cancers are checkpoint inhibitors, vaccines, cell-based antibodies, and oncolytic viruses [288]. Here, we will review the antibody- and cell-based methods. In cancer, immunotherapy refers to either activating or rescuing an anti-tumor immunogenic response or suppressing an undesirable immune dysfunction state to destroy existing cancer cells or control the progression. This is often achieved by identifying and targeting specific proteins usually expressed only by malignant cells using antibody- or cell-based methods. Monoclonal or bispecific antibodies are designed against the malignant cell proteins to destroy cancer cells while leaving healthy cells untouched. Similarly, healthy immune cells (mainly T- or NK-cells) derived from the patient are engineered in the laboratory to express specific antibodies or peptides against cancer cell-specific proteins and termed chimeric antigen receptor (CAR) cells, which can home onto cancer cells and kill them via cell-mediated cytotoxic responses. The patient’s immune function can also be rescued using specific recombinant cytokines and growth factors. Immunotherapy can be used as a monotherapy or in combination with a cytotoxic/chemotherapeutic drug to eliminate cancer cells while minimizing off-target effects.

Bispecific T-cell engagers (BiTEs) have two specific variable region chains—one for the immune cell receptor and one for the cancer-specific target antigen—to improve specificity and reduce off-target action.

**Figure 6:**
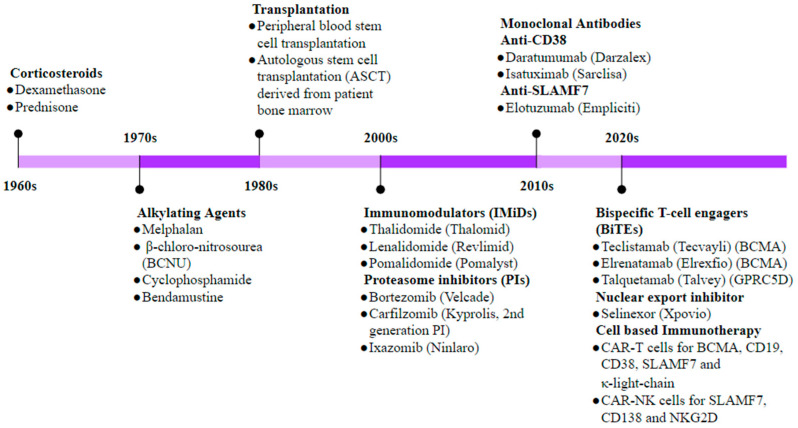
Timeline of therapeutic developments in MM. Historically, corticosteroids were the first drugs to be used in MM treatment, and in the late 1970s, BM stem cell transplantation became one of the most promising treatments in eligible patients. Discovery of the proteasome inhibitor (PI) bortezomib was a significant advancement in MM treatment and remains the standard first-line therapy in most MM regimens today. The last decade has witnessed significant research into targeting the dysregulated immune landscape as well as myeloma cell-specific markers through development of antibody- and cell-based immunotherapeutic approaches.

MM’s standard-of-care treatment has remained relatively the same since autologous stem cell transplantation (ASCT) was developed in the 1980s and, later, PIs in the 2000s (Figure 6). MM remains an incurable disease with high relapse and drug resistance rates [289,290,291,292,293]. Cell- and antibody-based immunotherapy are emerging as invaluable tools in MM treatment, with clinical trials showing great promise. B-cell maturation antigen (BCMA) and SLAMF7 are the two novel targets in MM immunotherapy. BCMA is an important member of the TNFR superfamily expressed by all malignant MMPCs and required for survival, proliferation, and myeloma progression. At the same time, SLAMF7 is thought to play a role In BM stromal interactions with MMPCs to promote survival and is also highly expressed by all stages of MMPCs [294,295]. The landmark advancement in MM therapy was the development and approval of the monoclonal antibodies elotuzumab (anti-SLAMF7) and daratumumab (anti-CD38) in 2015 for both monotherapy and combination forms, particularly in the case of RRMM [296,297,298,299,300,301]. The first anti-BCMA CAR-T cells were made by lentiviral vector-mediated transfection in 2013 using a single-chain variable fragment from mouse anti-BCMA antibody combined with hinge and transmembrane regions of human CD8α, CD3ζ T-cell activation domain, and a costimulatory molecule (CD28), and the first clinical trial took place in 2016 to show potent cytotoxicity in refractory MM [302,303]. Since then, a wide array of immunotherapies have been developed, including chimeric antigen receptor-T (CAR-T) cells, bispecific antibodies, antibody-drug conjugates, and immune checkpoint inhibitors [262,289,290]. A combination of the PI3K inhibitor with BCMA was used to develop the Bb2121 CAR T-cell therapy, designed to improve the memory phenotype for RRMM. In clinical trials, this showed minimal residual disease (MRD) negativity and median progression-free survival (PFS) of 17.7 months [304]. LCAR-B38M, a dual epitope anti-BCMA-targeting CAR-T therapy, underwent a Phase 1 clinical trial and showed a median duration of response (DOR) of 16 months, median PFS of 15 months, and median PFS for patients achieving CR of 24 months [305]. The CARAMBA Phase 1/2A clinical trial employs CAR-T cells targeting SLAMF7, utilizing a novel virus-free transient mRNA expression system to minimize the incidence of cytokine release syndrome (CRS) in the first European virus-free CAR-T clinical trial [306]. CAR-NK cell therapy has also been developed against SLAMF7, which was shown to eliminate human MM cells in a mouse xenograft model and is currently under phase 1 clinical trial evaluation (NCT03710421) [307]. A majority of MMPCs express CD56—required for MM cell peripheral blood egress and setup of extramedullary disease—which is also an emerging CAR cell therapy target showing positive results in a mouse model and currently in clinical trials (NCT03473496, NCT03271632) [308,309]. Another exciting target is CD19, which is rarely expressed by MMPCs but could potentially be expressed by “stem” clones in some patients. It has also been tested in two clinical trials with no conclusive results [308]. Other important targets in CAR cell therapy are CD138, κ-light chain, G-protein coupled receptor (GPRC) 5D, NKG2D, and New York Esophageal Squamous Cell Carcinoma (NY-ESO)-1, also being investigated in several clinical trials [308]. Table 3 lists successful ongoing and completed clinical trials for immunotherapy in MM at various stages of the disease. An anti-BCMA antibody, erlanatamab, is currently in a Phase 1 dose escalation clinical trial (NCT03269136) for RRMM post-PI or anti-CD38 treatment over an 8.1 month follow-up, with 67% of participants showing Grade 1 or 2 CRS, 31% complete remission (CR), 64% overall response rate (ORR), and 91% probability of event-free status at 6 months [310]. Several other anti-BCMA BiTEs and BsAbs are in various stages of clinical trials, and more targets are being explored, including CD138, CD38, CD19, SLAMF7, Fc receptor-like 5 (FcRL5), CS1-NKG2D, GPRC5D, and NY-ESO-1 [311].

## 5. Conclusions

This review emphasizes the intricate, dynamic network of cellular crosstalk between MMPCs and their BMME. All the components of the normal BM, including the ECM, blood vessels, osteo-adipo lineage cells, supporting cells, and the hematopoietic progeny, are eventually transformed by the myeloma cells to abet their destructive march through the bone and eventually into the bloodstream. These cells not only inhibit MM cell apoptosis, whether autonomous or drug-induced, but also contribute to the development of osteolysis. Therefore, it is imperative to consider these cellular interactions in the pursuit of innovative MM treatment approaches. A deeper understanding of the interactions among healthy cells within the BMME will provide valuable insights into how MM disrupts this milieu. Ongoing research efforts are focused on the development of novel MM treatment targets. In recent years, the introduction of agents targeting MM cells and their interactions with the BMME, such as proteasome inhibitors (e.g., bortezomib and carfilzomib) and immunomodulatory agents (e.g., thalidomide, lenalidomide, and pomalidomide), has significantly reduced mortality in MM patients. However, further elucidation of the molecular mechanisms governing MM-bone interactions and the MM niche is necessary to identify novel approaches that can positively impact bone disease and MM tumor progression.

SCTS studies in MM have greatly enhanced our understanding of the tumor microenvironment and the interplay of immune cells, MMPCs, and the BME. The studies reviewed here represent only the beginning of the ever-growing breadth of single-cell sequencing analysis. To truly impact clinical outcomes through genomic medicine, it is imperative to elucidate MM complexity through extensive large-cohort analyses to predict treatment response and improve overall prognosis. Multi-omics investigations, which integrate genetic sequencing with phenotypic assessments, promise to provide a comprehensive overview of dysregulation within various cell types and compartments throughout the disease continuum, spanning from MGUS and SMM to full-blown MM. Some limitations faced during single-cell analysis include the need for pure populations of cells for studies dealing exclusively with profiling PCs, and this becomes difficult in early stages such as MGUS or SMM, where the PC burden would be low and distinguishing malignant and non-malignant PCs becomes challenging as there would be some overlap between surface markers. Ultimately, this wealth of data will facilitate the molecular profiling of tumors across all disease stages, offering a dual clinical benefit for patients: precision medicine and precision diagnosis. While precision medicine has been strongly emphasized, aimed at tailoring therapies to individual MM patients based on molecular profiles, precision diagnostic strategies are also in development. These strategies target asymptomatic individuals with MGUS and SMM, offering early interventions to impede progression to MM. Harnessing the genomic insights accumulated over the past decade will lead to innovative approaches that can potentially enhance outcomes for MM patients.

Although great strides have been made in cancer immunotherapy, the burden of off-target effects, graft versus host disease, and cytokine release syndrome remains a significant cause of mortality post-treatment. MM remains an incurable disease with a complex pathophysiology. The interplay between MMPCs and the bone marrow (BM) microenvironment plays a pivotal role in MM’s pathogenesis and progression. Current treatment standards and most active anti-MM drugs target both MM cells and the BM microenvironment. By directing efforts toward these diseased cells and their microenvironment, novel treatment strategies may be identified to improve outcomes for individuals grappling with this incurable blood cancer.

## Figures and Tables

**Table 1:** Staging of myeloma based on myeloma-defining events, cytogenetic abnormalities, and prognosis [27,28,29,30,31,32,33,34,35,36,37,38]. * CRAB criteria defined by the International Myeloma Working Group (IMWG) refer to organ involvement based on hyperCalcemia (serum calcium > 10.5 mg/dL), Renal impairment (serum creatinine > 2 mg/dL), Anemia (hemoglobin < 10 g/dL), and Bone lesions (osteolysis or osteoporosis on any skeletal examination). LR = Low Risk, SR = Standard Risk, IR = Intermediate Risk, HR = High Risk, IS = Improved Survival, AP = Adverse Prognosis for progression-free/overall survival.

Myeloma Stage	Clinical Parameters	Cytogenetic Abnormalities	Prognosis
MGUS	Serum M-protein ≤ 30 mg/l, <10% BM clonal PC, no organ damage evidence (CRAB *)	Hyperdiploidy (HD) (chromosomes 3, 5, 7, 9, 11, 15, 17)	SR
		IgH translocations (IgHT)—t(11;14), t(4;14), t(6;14), t(14;16) and t(14;20) and involved partner genes—4p16: *FGFR3/MMSET*, 11q13: *CCND1*, 16q23: MAF, 6p21: *CCND3*, 20q11: *MAFB*	HR, AP
		monosomy 13/del(13q)	SR/IR
SMM	Serum M-protein ≥ 30 mg/l (IgG or IgA), >10% BM clonal PC, no organ damage evidence (CRAB)	MGUS abnormalities	SR
		MUGUS + secondary abnormalities—monosomy17/del(17p) (gene P53), gain 1q21	HR
MM	Serum M-protein ≥ 30 mg/l (IgG or IgA), >10% BM clonal PC, and organ damage evidence (CRAB)	HD, HD (chromosomes 3, 5) + gain 5q31	IS
		Hypoploidy, IgHT + non-HD	HR, AP
		del(17p), gain 1q21 (gene *CSK1B*), *MYC* translocations, del(1p)	AP
Extramedullary MM (not including solitary/bone plasmacytoma)	MM criteria and involvement of skeleton or soft tissue or lymph node(s), BM-independence, drug resistance	del(17p13), del(13q14), *MYC*-over-expression, t(4;14)	AP
		Mutations in *TP53, RB1, KRAS, FAK*	HR
		del(17p) + non-HD	AP
RRMM	Reappearance of M-protein, ≥5% BMPCs, new lytic bone lesions and/or soft tissue plasmacytomas, increase in size of residual bone lesions, and/or development of hypercalcemia > 11.5 mg/dL not attributable to another cause	del(17p), t(4;14) or t(14;16)	HR, AP
		gain 1q21	AP
		t(4;14): overexpression of *FGFR3*, t(14:16): overexpression of *c-maf*, t(14:20): overexpression of *c-maf*, del(17p): deletion of *p53*	AP

**Table 2:** Recent single-cell sequencing studies in MM exploring the role of immune cells and the BMME in myelomagenesis, progression, and drug response. NA = not available.

Disease Stage	Type of Samples and Number of Cells	Number of Samples	Type of Sequencing	Key Findings	Year and Reference
MGUS, SMM, MM, primary light chain amyloidosis (AL)	PCs from peripheral blood (3540) and BM (20,586)	MGUS = 7, SMM = 6, MM = 12, AL = 4, controls = 11	scRNA-seq	Use of single cell transcriptomics for prognostic prediction and personalized therapy based on unique subclonal structures within individuals, detection of tumor cell populations in MRD stages matching those from active MM.	2018 [279]
5TGM1 murine myeloma	NA	NA	scRNA-seq	Identification of myeloma-specific transcriptome signature enriched for genes associated with immune function and myeloid cell differentiation, loss of dormancy related gene expression such as *AXL*	2019 [280]
MGUS, SMM	Patient BM aspirates, 19,000 CD45+/CD138− cells	MGUS = 5, LR-SMM = 3, HR-SMM = 8, NDMM = 7, healthy donors = 9	scRNA-seq	Immune profile-based patient risk stratification: increased NK cell abundance in early stages, loss of cytotoxic T cells in SMM, MHC-II dysregulation in CD14+ monocytes causing T-cell suppression.	2020 [281]
NDMM to relapse follow-up samples	Patient BM aspirates, CD138+ sorted and unsorted cells: 17,267 PCs and 57,719 immune cells	14 patients across stages for a total of 29 samples	scRNA-seq and 10x WGS	Three distinct patterns identified with stability from precancer to diagnosis and gain or loss from diagnosis to relapse involving B-cell type PCs, inflammation regulated gene expression changes in *IL6* and *IL1B*	2021 [282]
Across MM stages	Patient BM aspirates. CD138+ and CD138− sorted BM cells	18 patients across stages	scRNA-seq, CyTOF, and CITE-seq	Advanced stage MM patients had reduced CD4+ T/CD8+ T cells ratio, overexpression of *RAC2* and *PSMB9* observed in NK cells of progressors compared to non-progressors, rapid progression-specific markers identified for MM	2022 [283]
MM before and after 2 cycles of bortezomib-cyclophosphamide-dexamethasone (VCD) treatment	Patient and control BM and peripheral blood samples, 25,231 plasma cells and 216,209 immune cells	MM patients = 10, healthy controls = 3	scRNA-seq	In response to drug treatment, reduced unfolded protein response and metabolic signaling, increased stress and immune-reactive signaling; reduced checkpoint molecules expression, T-cell, NK cell and monocyte exhaustion in MM indicating immunosuppression in the BMME and related to poor prognosis	2023 [284]
Across MM stages	NA	NA	scRNA-seq	Progression associated with immunosuppressive tumor microenvironment, with increased levels of exhausted CD8+ T-cells, NK cells and reprogrammed TAMs showing both M1 and higher M2 phenotypes with impaired phagocytic activity demonstrated in vitro	2023 [285]
NDMM followed by 2 cycles of VCD	BM and peripheral blood samples, DCs = 1429, monocytes = 42,464	MM patients = 10, healthy volunteers = 3	scRNA-seq	Dysfunctional conventional DC2 (cDC2), mono-DC, and intermediate monocytes through downregulation of interferon regulatory factor (IRF)-1 signaling with reduced antigen presentation capacity in MM compared to controls	2023 [286]

**Table 3:** Ongoing immunotherapy clinical trials for MM {adapted from the NCBI Clinical Trials Registry. Source: [312]}.

NCT Number	Study Title	Conditions	Targets	Type of Immunotherapy	Expected Outcome	Phases
NCT00090493	Study of MAGE-A3 and NY-ESO-1 Immunotherapy in Combo with DTPACE Chemo and Auto Transplantation in Multiple Myeloma	MM	Myeloma cells expressing MAGE-A3 and NY-ESP-1 proteins	Peptide vaccine against MAGE-A and NY-ESP-1	Generation of anti-myeloma T cells to kill myeloma cells	Phase 2, Phase 3
NCT00006244	Melphalan, Peripheral Stem Cell Transplantation, and Interleukin-2 Followed by Interferon Alfa in Treating Patients with Advanced Multiple Myeloma	Refractory MM, Stage I, Stage II and Stage III MM	Activating patient WBC response	Recombinant IL-2 (Aldsleukin), IFN-α	Stimulate patient WBCs with IL-2 to kill myeloma cells and arrest proliferation with IFN-α	Phase 2
NCT03525678	A Study to Investigate the Efficacy and Safety of Two Doses of GSK2857916 in Participants with Multiple Myeloma Who Have Failed Prior Treatment with an Anti-CD38 Antibody	MM	BCMA on myeloma cells	Antibody-drug conjugate (ADC)—belantamab mafodotin	Anti-BCMA antibody belantamab will target myeloma cells and the conjugate mafodotin is a cytotoxic agent to effect cell cycle arrest and ADCC of myeloma cells	Phase 2
NCT02336815	Selinexor Treatment of Refractory Myeloma	MM	Exportin-1 (XPO-1)—overexpressed in myeloma cells	Inhibitor of nuclear export	Arrest cell cycle and proliferation of myeloma cells by blocking nuclear to cytoplasmic transport and signaling	Phase 2
NCT03958656	T-cells Expressing an Anti-SLAMF7 CAR for Treating Multiple Myeloma	MM, plasma cell (PC) MM	SLAMF7	Anti-SLAMF7 CAR-T cell	Targeting myeloma cell SLAMF7 using CAR-T cell and T cells along with a stop gene to limit toxicity	Phase 1
NCT03602612	T Cells Expressing a Novel Fully Human Anti-BCMA CAR for Treating Multiple Myeloma	MM, PCMM	BCMA on myeloma cells	Anti-BCMA CAR-T cells	Patient- derived T-cells cultured to express anti-BCMA CAR to kill BCMA expressing myeloma cells	Phase 1
NCT02215967	Study of T Cells Targeting B-Cell Maturation Antigen for Previously Treated Multiple Myeloma	PCMM, MM	BCMA on myeloma cells of treated patients	Anti-BCMA CAR-T cells	Patient- derived T-cells cultured to express anti-BCMA CAR to kill BCMA expressing myeloma cells	Phase 1
NCT03338972	Immunotherapy with BCMA CAR-T Cells in Treating Patients with BCMA Positive Relapsed or Refractory Multiple Myeloma	Recurrent PCMM, refractory PCMM	BCMA on myeloma cells of relapsed or refractory MM patients	Autologous Anti-BCMA-CAR-expressing CD4+/CD8+ T-lymphocytes	Patient- derived T-cells cultured to express anti-BCMA CAR to kill BCMA expressing myeloma cells	Phase 1
NCT00566098	Activated White Blood Cells with ASCT for Newly Diagnosed Multiple Myeloma	MM and PC neoplasm	Newly diagnosed MM cells	Activated patient-derived WBCs	Use patient-derived, cultured WBCs to study MM cell cytotoxicity and side effects	Phase 1, Phase 2
NCT01245673	Combination Immunotherapy and Autologous Stem Cell Transplantation for Myeloma	MM	Myeloma cells	MAGE-A3 vaccine + activated T-cells	To test if this combination: (i) provides “immunity” against myeloma cells and (ii) prevents progression	Phase 2
NCT00439465	Adoptive Cellular Immunotherapy Following Autologous Peripheral Blood Stem Cell Transplantation (APBSCT) for Multiple Myeloma	Transplant eligible MM patients	Myeloma cells in post-APBSCT patients	Cytotoxic T-cells + IL-2 + recombinant GM-CSF	Combination of Cytotoxic T-cells + IL-2 + recombinant GM-CSF to enhance anti-tumor immune reconstitution and improve outcome of MM patients.	Phase 2
NCT05652530	Clinical Study of the Safety and Efficacy of BCMA CAR-NK	MM	BCMA on myeloma cells	Anti-BCMA CAR-NK cells	Targeting myeloma cells expressing BCMA using CAR-NK cells in relapsed/refractory MM patients	Early Phase 1
NCT03940833	Clinical Research of Adoptive BCMA CAR-NK Cells on Relapse/Refractory MM	MM	BCMA on myeloma cells	Anti-BCMA CAR-NK 92 cells	To kill myeloma cells expressing BCMA using NK 92 cells in MM patients	Phase 1, Phase 2
NCT05182073	FT576 in Subjects with Multiple Myeloma	MM	BCMA on myeloma cells	FT576 (Allogeneic BCMA CAR-NK cells)	FT576 as monotherapy and in combination with the monoclonal antibody daratumumab in MM	Phase 1
NCT06045091	To Evaluate the Safety and Efficacy of Human BCMA Targeted CAR-NK Cells Injection for Subjects With R/R MM or PCL	MM, PC leukemia	BCMA on myeloma cells	Anti-BCMA CAR-NK cells	Targeting myeloma cells expressing BCMA using CAR-NK cells in MM and PC leukemia patients	Early Phase 1
NCT05008536	Anti-BCMA CAR-NK Cell Therapy for the Relapsed or Refractory Multiple Myeloma	Refractory MM	BCMA on myeloma cells	Umbilical and cord blood derived, anti-BCMA engineered CAR-NK cells	Targeting malignant myeloma cells expressing BCMA using engineered CAR-NK cells in refractory MM patients	Early Phase 1

## Data Availability

Not applicable.
